# Mental health needs of heart and lung transplant recipients in Ireland using the Stanford Integrated Psychosocial Assessment for Transplantation (SIPAT) tool

**DOI:** 10.1192/j.eurpsy.2023.855

**Published:** 2023-07-19

**Authors:** K. Corrigan, G. Crudden, B. Mohammed, R. Duffy, A. Doherty, Z. Azvee, A. Clarke

**Affiliations:** 1 Liaison Psychiatry, The Mater Misericordiae University Hospital; 2 Perinatal Psychiatry, Rotunda Hospital; 3 School of Medicine, University College Dublin; 4 Adult Psychiatry, St Vincent’s Hospital Fairview; 5 Liaison Psychiatry, The Mater Misericordiae Hospital, Dublin, Ireland

## Abstract

**Introduction:**

International guidelines recommend that prospective organ transplant patients receive a psychosocial assessment to optimise outcomes. There is limited consensus regarding the criteria for psychosocial evaluation. The Stanford Integrated Psychosocial Assessment for Transplantation (SIPAT) tool was developed to enhance the pre-transplant psychosocial workup. The Mater Hospital is the National Centre for heart and lung transplantation in Ireland. The consultation-liaison psychiatry (CLP) service provides screening of pre-transplant candidates using a biopsychosocial assessment, SIPAT and cognitive screening tools. Post-transplant patients are reviewed on a referral basis.

**Objectives:**

To identify the psychosocial needs of heart and lung transplant recipients and CLP service input over a one year period.

**Methods:**

A review of all heart and lung transplant recipients between January 1^st^ and December 31^st^ 2021 was conducted. The following data were recorded: demographics, pre-existing mental illness, SIPAT scores, and referral to the CLP service within six months of transplantation.

**Results:**

Twenty-eight individuals received a heart/lung transplant in 2021 (7 heart, 19 lung, 1 heart &liver, 1 heart & lung). Prior to transplant 50% (14/28) had a pre-existing mental health diagnosis, 7% (2/28) had attended a psychiatrist, and 28.6% (8/28) were on psychotropic medication. SIPAT scores were available for 20 patients. The overall mean SIPAT score was 10.8 (SD 6.1). The subscales were as follows: Patient Readiness, mean 3.2 (SD 1.7); Social Support System, mean 2.1 (SD: 1.8); Psychological Stability & Psychopathology, mean 1.6 (SD 2.7); and Lifestyle & Substance Misuse, mean 3 (SD 1.5). Based on SIPAT scores, 20% (4/20) were *excellent* candidates, 75% (15/20) were *good* candidates and 1 (1/20) was *minimally acceptable.* Pre-existing mental illness was associated with higher total SIPAT scores (p=0.013) and higher scores on the psychological stability subscale (p=0.032). Post-transplant, 50% (14/28) were referred for psychological support and 21.4% (6/28) were referred to the CLP service. A further 10.7% (*n*=3) were attending CLP prior to transplant. Referrals to CLP occurred (median) 13 days (range 1-275) post-transplant surgery. The reasons for attending CLP were anxiety (5/9), delirium (3/9) and mood (1/9).

**Conclusions:**

SIPAT can be a valuable tool for use in the pre-transplant workup to help identify those that will require intensive psychosocial support post- transplant.

**Disclosure of Interest:**

None Declared

